# Below the Belt: A Case of an Atypical, Large Perineal Lump

**DOI:** 10.7759/cureus.114867

**Published:** 2026-08-20

**Authors:** Rohit Deshpande, Nivetha Ambalavanan, Aditya P Sharma

**Affiliations:** 1 Urology, Post Graduate Institute of Medical Education and Research, Chandigarh, IND; 2 Histopathology, All India Institute of Medical Sciences, Mangalagiri, IND

**Keywords:** epidermal inclusion cyst, giant epidermoid cyst, magnetic resonance imaging (mri), perineal swelling, perineum reconstruction

## Abstract

Perineal lumps can cause significant distress and functional impairment, often presenting diagnostic challenges due to a wide range of differential diagnoses, including abscesses, median raphe cysts, benign teratomas, and other rare soft-tissue lesions. While epidermoid cysts are common, benign cutaneous lesions frequently encountered on the neck, trunk, or face, their occurrence in the perineum is a rarity. When present, they are often secondary to mechanical pressure, minor trauma, or previous surgical interventions.

A 30-year-old gentleman presented with an insidiously growing, painless perineal swelling measuring approximately 8 x 8 x 6 cm that significantly interfered with his ability to sit and perform daily activities. Clinical examination revealed a superficial, well-defined, cystic, and mobile mass with no signs of inflammation, toxemia, or gastrointestinal involvement. An MRI was utilized to evaluate the mass, demonstrating a well-marginated midline lesion clearly separated from the testes, anal sphincters, and surrounding musculature. Based on these findings, the patient underwent complete surgical excision of the mass via an elliptical vertical incision.

The mass was successfully excised *in toto*, with careful dissection ensuring completeness of removal of the cyst wall. Macroscopic examination of the resected tissue's cut section revealed thick, inspissated sebaceous material. Subsequent histopathological evaluation demonstrated a dermal-based cyst lined by stratified squamous epithelium with an intact granular layer and lamellated keratin flakes with absent adnexal structures, definitively confirming an epidermal inclusion cyst.

Although infrequent in the perineum, an epidermal inclusion cyst should be amongst the differential diagnoses of slow-growing, atypical perineal swellings. Advanced imaging techniques like MRI are valuable for preoperative planning, but complete surgical extirpation remains the definitive standard of care. Removing the entire cyst wall successfully relieves patient discomfort, provides necessary tissue for histopathological diagnosis, and effectively prevents future recurrences.

## Introduction

Perineal lumps are an important cause of significant discomfiture, which can lead to difficulty in sitting, performing daily activities in the seated position, and a constant sense of uneasiness for the patient. The differentials of a large perineal lump include a perineal abscess, a median raphe cyst [1], a benign teratoma, a Bartholin’s cyst, vulval cysts, an ectopic testicle [2] with hydrocele, a congenital lipoma [3], a leiomyoma [4], thrombosed hemorrhoids or intersphincteric cysts [5] in rare cases, or the very rare angiomyxoma [6].

While a perineal abscess can often be associated with fever, a prostatic abscess, or a fistula-in-ano, other causes of a perineal lump often do not cause toxemia and can be asymptomatic initially. The patient may only present in later stages, when the swelling is slow-growing or becomes large enough to interfere with daily activities like sitting or driving.

While prostatic abscesses are relatively rarer, fistula-in-ano is a relatively common finding in the general population. Hair-bearing skin typically harbors pathological lesions such as epidermoid cysts, which arise from the infundibulum or the upper portion of a hair follicle. While the face, scalp, and trunk are relatively common sites for large epidermoid cysts, the perineum is an uncommon location. Although superficially located and thin-walled epidermoid cysts are prone to rupture, longstanding epidermoid cysts, which have enlarged in size and become organized, become hardened by inspissated secretions and therefore do not rupture easily.

The patient in the index case presented to us when the perineal swelling had become significantly large, i.e., large enough to interfere with his activities of daily living. Perineal epidermoid cysts are considered extremely rare, documented primarily through isolated individual case reports and small clinical series in medical literature rather than large epidemiological studies.

## Case presentation

**Figure 1:**
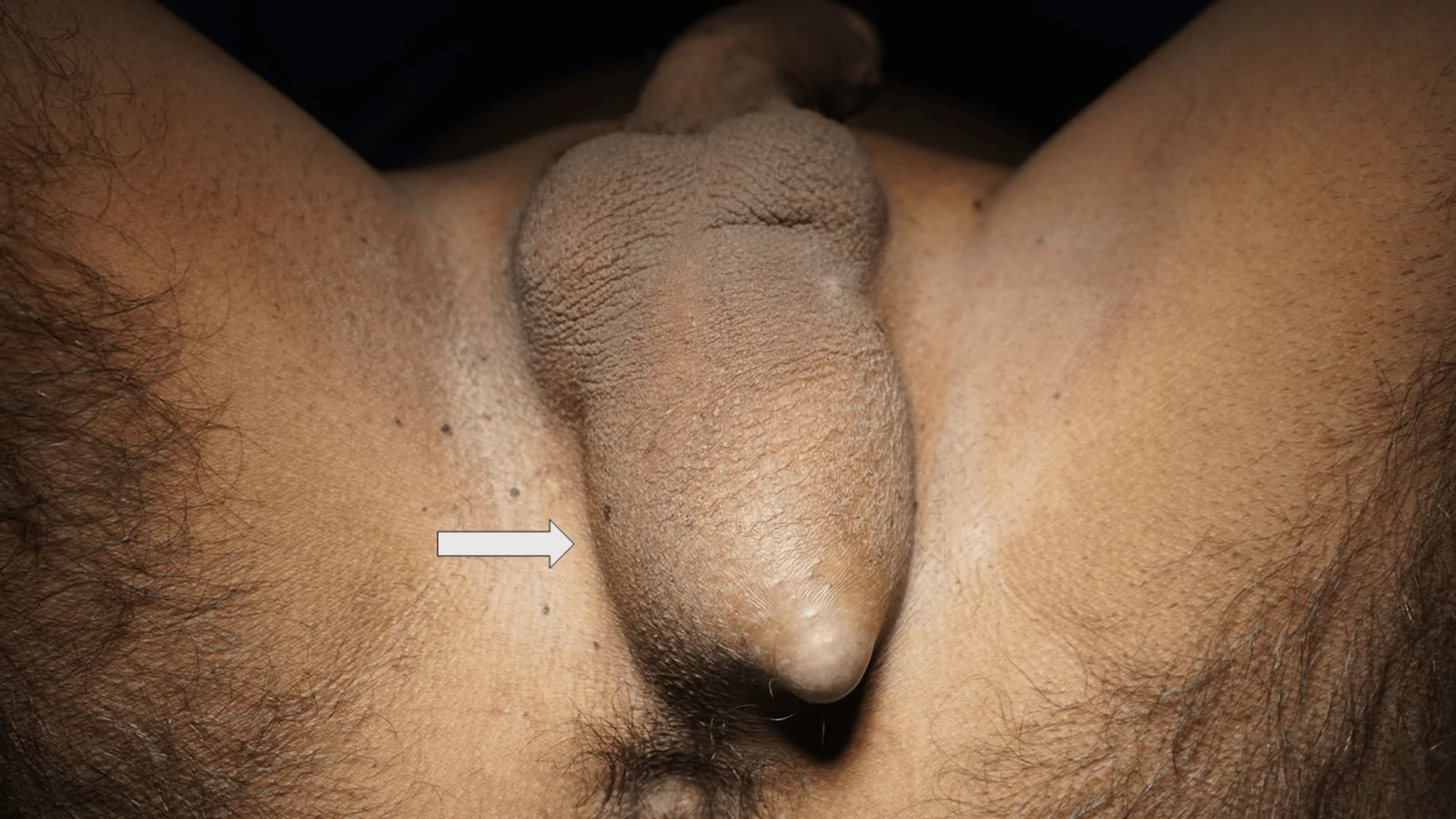
White arrow points to the perineal swelling, causing slight upward displacement of the testes, with no signs of inflammation.

A 30-year-old gentleman presented to the outpatient department with an insidiously growing perineal swelling that was painless, interfered in his daily activities like sitting, was not associated with fever or any signs of toxemia, and was not associated with any gastrointestinal symptoms like constipation/bleeding per rectum/past history of fistula-in-ano. On examination (Figure 1), the swelling was superficial in location, approximately 8 x 8 x 6 cm in dimensions, free from the anal margin, having well-defined borders, cystic in consistency, non-transilluminant, freely mobile, and not showing any pus points or any other signs of inflammation on the overlying skin and surrounding tissues.

**Figure 2:**
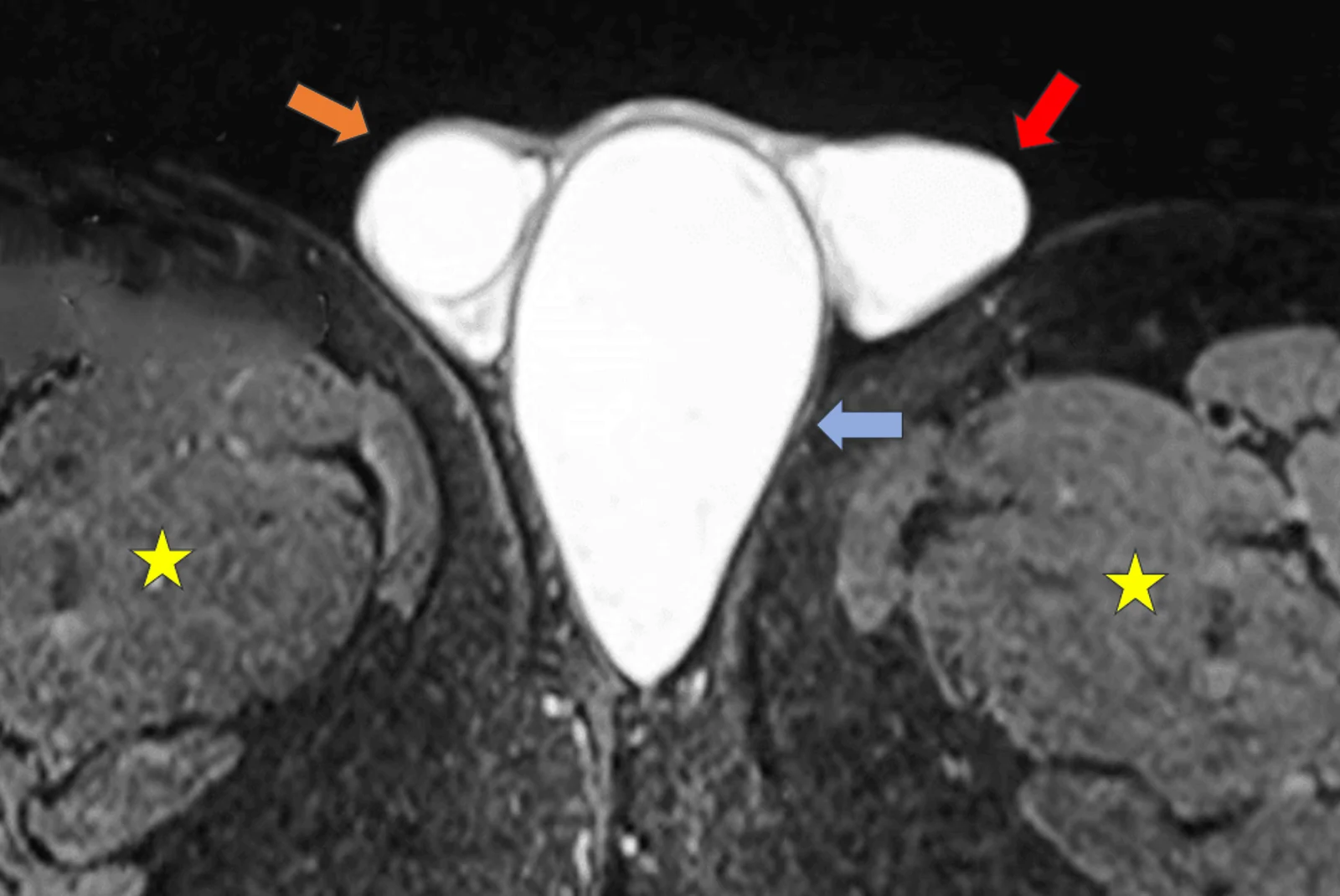
MRI image of the perineal lump in the midline, separate from the testes and surrounding muscular structures/anal sphincters Orange arrow: Right testis; Red arrow: Left testis; Blue arrow: Midline perineal swelling, appearing hyperintense on T2 -weighted imaging; Yellow star: Thigh musculature

The testicles were displaced slightly cranially, were separately felt, and were normal. Per-rectal digital examination was essentially normal and did not reveal any internal openings of a suspected fistula-in-ano. On MRI (Figure 2), the lump appeared to be well-marginated, with homogeneous contents.

**Figure 3:**
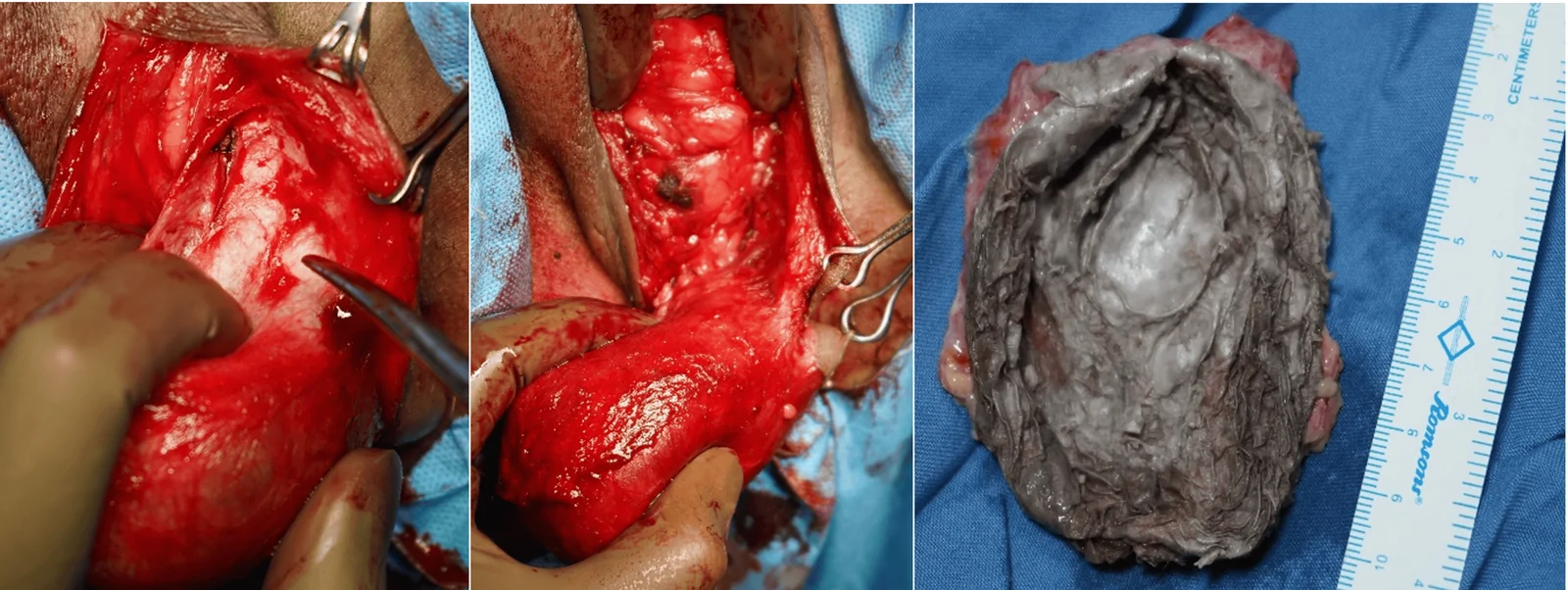
Intraoperative images and gross appearance of the specimen Intraoperative images show the separation of the mass from surrounding perineal muscles. The cyst wall was removed in its entirety to obviate the risk of recurrence. The cut section of the mass revealed inspissated sebaceous material.

**Figure 4:**
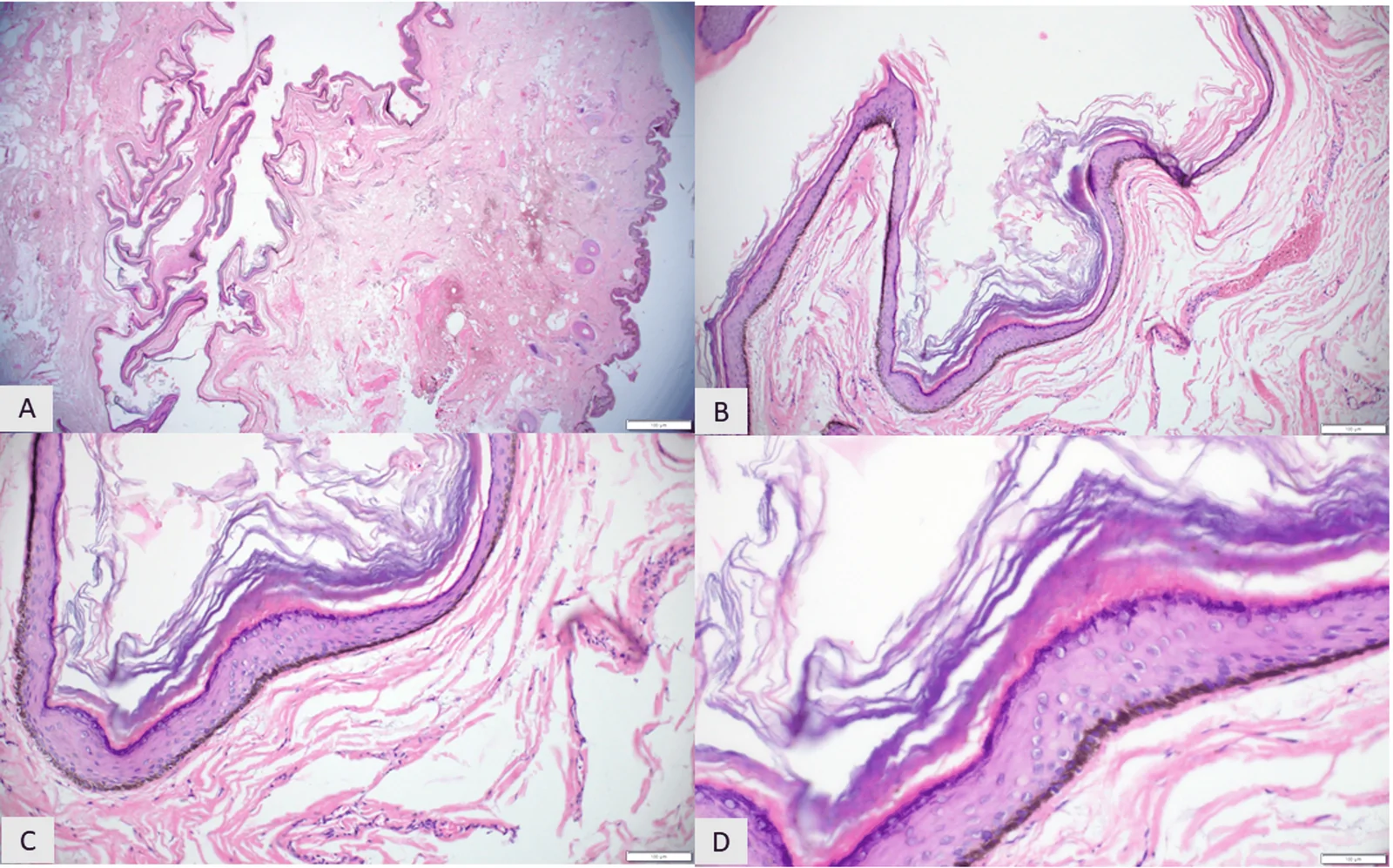
Histopathological appearance of epidermal inclusion cyst A: Sections show a dermal-based cyst (H&E, 20x), B: The cyst is lined by stratified squamous epithelium with lamellated keratin flakes, C: H&E 200x, D: Intact granular layer (H&E 400x) with absence of adnexal structures

A plan for surgical excision was made. An elliptical vertical incision was made. The lump was carefully separated from surrounding structures and excised in toto. A cut section of the lump revealed thick sebaceous material (Figure 3). Histopathological examination revealed features of an epidermal inclusion cyst (Figure 4).

## Discussion

Frequently encountered in clinical practice, an epidermoid cyst is a common, benign, cutaneous lesion that typically presents as a slow-growing, asymptomatic nodule. While they are predominantly found in areas rich with pilosebaceous units, such as the neck, trunk, and face, they are notably rare in the perineum and lower extremities. Although these cysts can theoretically occur at any point in a patient's life, epidemiological data show they most frequently develop during early to middle adulthood, specifically peaking in the third or fourth decades of life. Furthermore, there is a distinct gender predilection; they are roughly twice as common in males as in females, a variance often attributed to differences in sebaceous gland activity, hormones, or occupational exposures.

The underlying pathogenesis of epidermoid cyst production involves the abnormal encapsulation and proliferation of epidermal cells within the deeper dermis. This process has been linked to several systemic and environmental causes, such as chronic exposure to ultraviolet (UV) radiation, smoking, infections like the human papillomavirus (HPV), and blunt trauma. However, when evaluating the rare instances of these cysts in the perineal region, the etiology is often distinct. In this area, the main contributing elements are usually iatrogenic (medically induced) or mechanical. Constant mechanical pressure, repetitive slight trauma, or direct epidermal implantation from surgical operations, such as needle biopsies, episiotomies, or routine pelvic surgeries, can inadvertently push surface epithelial cells into deeper subcutaneous tissues, where they multiply and form a keratin-filled cyst [7].

While a superficial epidermoid cyst can often be diagnosed via physical examination and patient history, deeper or atypically located cysts require radiological evaluation. When it comes to narrowing down the differential diagnoses in a broad range of possible soft tissue lesion diagnoses, such as distinguishing them from lipomas, dermoid cysts, or malignant tumors, MRI is the preferred advanced imaging modality. While ultrasound is frequently used as a first-line tool, MRI provides superior soft-tissue resolution, which is essential for accurate pre-surgical planning and defining the lesion's relationship with surrounding anatomical structures.

On an MRI scan, epidermoid cysts exhibit highly characteristic radiological features. They typically appear as sharply marginated, well-defined mass lesions that demonstrate hypointensity on T1-weighted imaging, which reflects their dense internal makeup of desquamated keratin and cellular debris. Conversely, they show striking hyperintensity on T2-weighted and fluid-sensitive sequences due to their cystic nature and high internal water content. Following the administration of intravenous contrast, these cysts classically display a thin, smooth peripheral post-contrast enhancement [8]. This specific 'rim' enhancement pattern occurs because the fibrous capsule surrounding the cyst contains blood vessels, while the central core remains entirely avascular. Additionally, advanced MRI techniques like diffusion-weighted imaging (DWI) often reveal restricted diffusion, a hallmark finding that gives radiologists high confidence in the diagnosis.

## Conclusions

Complete surgical extirpation remains the standard treatment of choice for managing large perineal cystic lesions. Due to the high-friction, weight-bearing nature of the perineal region, these masses often cause significant mechanical discomfort, pain during sitting, and a heightened susceptibility to secondary infections. Complete excision is essential to help alleviate the patient's debilitating symptoms while simultaneously preventing complications like spontaneous rupture. Furthermore, an intact surgical removal provides a definitive excisional biopsy for comprehensive histopathological diagnosis. This pathological evaluation is a critical step, as it firmly establishes the exact nature of the cyst and explicitly rules out rare but possible malignant transformations, such as squamous cell carcinoma, which cannot be adequately assessed through simple needle aspiration alone.
